# Wnt3a promotes in situ dentin formation through NKD1-MSX1 axis-mediated odontogenic differentiation of dental pulp stem cells

**DOI:** 10.1038/s41368-025-00406-3

**Published:** 2026-01-13

**Authors:** Haoran Du, Qiong Li, Chenchen Zhou, Junji Xu, Kang Gao, Zixiao Li, Yifan Xu, Ousheng Liu, Bing Li, Jianguang Xu, Jingsong Wang, Hideaki Kagami, Xianqi Li, Su Chen, Jian Zhou

**Affiliations:** 1 Department of International Medical Center, Beijing Stomatological Hospital, Capital Medical University, Beijing, China; 2 Beijing Laboratory of Oral Health, Capital Medical University, Beijing, China; 3 Laboratory of Tissue Regeneration and Immunology and Department of Periodontics, Beijing Key Laboratory of Tooth Regeneration and Function Reconstruction, School of Stomatology, Capital Medical University, Beijing, China; 4 Laboratory for Clinical Medicine, Capital Medical University, Beijing, China; 5 State Key Laboratory of Oral Diseases & National Clinical Research Centre for Oral Diseases & Department of Pediatric Dentistry, West China Hospital of Stomatology, Sichuan University, Chengdu, Sichuan China; 6 College of Stomatology, Chongqing Key Laboratory of Oral Diseases and Biomedical Sciences, Chongqing Municipal Key Laboratory of Oral Biomedical Engineering of Higher Education, Chongqing Medical University, Chongqing, China; 7 Xiangya Stomatological Hospital, Central South University, Changsha, Hunan China; 8 School and Hospital of Stomatology, Shanxi Medical University, Taiyuan, Shanxi China; 9 College & Hospital of Stomatology, Key Laboratory of Oral Diseases Research of Anhui Province, Anhui Medical University, Hefei, Anhui China; 10 Department of Dentistry and Oral and Surgery, Aichi Medical University, Nagakute, Aichi Japan; 11 Department of Oral and Maxillofacial Surgery, School of Dentistry, Matsumoto Dental University, Shiojiri, Japan; 12 Laboratory for Oral and General Health Integration and Translation, Beijing Tian tan Hospital, Capital Medical University, Beijing, China

**Keywords:** Stem-cell research, Regeneration

## Abstract

The functional regeneration of the dentin-pulp complex is pivotal for tooth preservation, yet the molecular mechanisms governing odontoblast differentiation remain poorly understood. In the current study, we revealed a distinct NKD1^+^ subpopulation exhibiting secretory odontoblast characteristics, which was specifically induced in dental pulp stem cells (DPSCs) by Wnt3a, but not by Wnt5a or Wnt10a through single-cell transcriptomic profiling. We then found that the NKD1^+^ subpopulation was functional conservation, which were consistently identified in the odontoblast layers of developing tooth germs in both murine and miniature pig models, as well as within the apical open area in human molars. This conserved spatial distribution and co-localization with *DSPP* strongly indicates that NKD1^+^ cells were active dentin-secreting odontoblasts. Analysis of gene regulatory networks using SCENIC identified MSX1 as a key transcription factor regulating the specification of NKD1^+^ lineage. Mechanistically, Wnt3a orchestrates a tripartite cascade: upregulating NKD1/MSX1 expression, triggering NKD1 membrane detachment, and facilitating direct NKD1-MSX1 interaction to promote MSX1 nuclear translocation. CUT&Tag analysis demonstrated MSX1 occupancy at promoters of odontogenic regulators, establishing its necessity for odontogenic gene activation. Murine pulp exposure models validated that Wnt3a-activated NKD1-MSX1 signaling significantly enhances reparative dentin formation. This study delineates an evolutionarily conserved Wnt3a-NKD1-MSX1 axis that resolves stem cell heterogeneity into functional odontoblast commitment, providing both mechanistic insights into dentin-pulp regeneration and a foundation for targeted regenerative therapies.

## Introduction

The pulp-dentin complex maintains tooth vitality through reparative dentin formation, stress response, and homeostatic regulation.^1^ Functional loss of this complex (e.g., irreversible pulpitis) often leads to post-endodontic tooth fractures, ultimately requiring extraction.^2^ Consequently, functional regeneration of the pulp-dentin complex represents a critical strategy for long-term tooth preservation. Odontoblasts, acting as the core units of this complex, govern the efficacy of reparative dentinogenesis via their lineage-commitment plasticity.^1^ Therefore, deciphering the molecular signaling driving this differentiation cascade is a pivotal focus in dental regenerative medicine.

Over the past decades, multiple dental mesenchymal stem cells—including dental pulp stem cells (DPSCs),^3,4^ stem cells from human exfoliated deciduous teeth (SHED),^5,6^ and stem cells from apical papilla (SCAPs)^7^—have been characterized for pulp-dentin complex regeneration. However, emerging evidence indicates that transplanted stem cells primarily generate disorganized osteoid-like tissues lacking polarized odontoblast layers and tubular dentin structures. These findings underscore the insufficiency of stem cell transplantation alone and highlight the necessity for targeted signaling activation to drive DPSCs differentiation and pulp-dentin complex regeneration.

The Wnt/β-catenin pathway plays critical regulatory roles in odontogenesis initiation, morphogenesis, and dentin formation.^8,9^ Our previous study demonstrated that localized Wnt3a delivery in minipig root canals promotes endogenous SCAPs homing and functional regeneration of the pulp-dentin complex, characterized by polarized odontoblast layers, tubular dentin, and neurovascular network reconstruction.^10^ While these findings establish canonical Wnt signaling as indispensable for pulp regeneration, the mechanisms by which heterogeneous stem cell subpopulations recognize, transduce Wnt signals, and execute reparative dentinogenesis remain unresolved.^11^

Notably, cumulative evidence suggests that only minor subsets of stem cells drive tissue regeneration. For instance, only a subset of SHEDs exhibit superior pulp regenerative capacity,^12^ and single-cell transcriptomic profiling has revealed distinct subclusters within primary dental pulp cells.^13^ Similarly, a study identified a Krt14^+^Ctsk^+^ osteoprogenitor population with dual epithelial-mesenchymal properties as the determinant of maxillary sinus floor lift-induced osteogenesis.^14^ Recently, a pivotal study discovered a population of multipotent CD24a^+^ stem cells within the mouse dental papilla, demonstrating that this subpopulation naturally resides in the dental papilla niche and can form pulp-like tissue in vivo.^15^ This finding substantiates the potential of single-population multipotent stem cell subsets to regenerate pulp-like tissues. However, while these studies have identified multipotent stem cell subpopulations in developing dental papilla suitable for pulp regeneration, adult stem cells typically exhibit reduced multipotency compared to their developmental counterparts.^16^ It remains unknown whether these adult stem cells can undergo Wnt signaling-induced differentiation into one or multiple subpopulations with pulp regenerative capabilities.

In this study, we reconfirmed the robust odontogenic induction capacity of Wnt3a and identified a previously uncharacterized stem cell subpopulation that specifically exhibits high expression of NKD1, a target gene of the canonical Wnt signaling pathway. This population was conserved across murine, porcine, and human pulp-dentin complexes and exhibited secretory odontoblast-like functionality. Regulatory network analysis further revealed MSX1-dependent programming of NKD1^+^ cells, with NKD1 directly interacting with MSX1 to promote its nuclear translocation and odontogenic activity. Critically, Wnt3a application at exposed pulp sites induced NKD1^+^ cell recruitment and sustained reparative dentin formation. Our findings establish Wnt3a-induced NKD1^+^ odontoblasts as key drivers of functional pulp-dentin regeneration, providing both mechanistic insights and therapeutic targets for clinical translation.

## Results

### Wnt3a effectively induces DPSC differentiation into mature odontoblasts

**Fig. 1:**
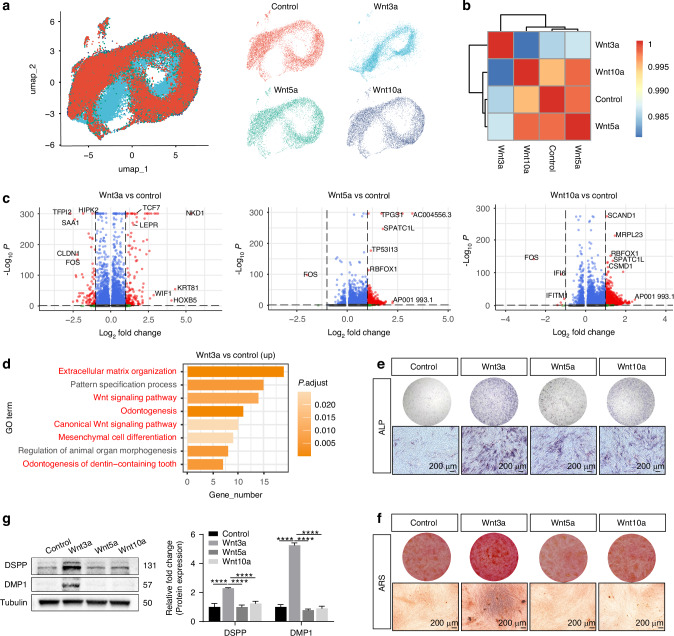
Wnt3a induces odontoblastic differentiation of DPSCs. **a** Uniform manifold approximation and projection (UMAP) of single-cell transcriptomic sequencing data from DPSCs treated with blank, Wnt3a, Wnt5a, and Wnt10a under odontogenic induction conditions. **b** The correlation heatmap of scRNA-seq datasets. **c** Volcano plot illustrating the differentially expressed genes (DEGs) of Wnt3a, Wnt5a, and Wnt10a-treated groups compared with control group. **d** Gene Ontology (GO) enrichment analysis of upregulated DEGs in Wnt3a-treated groups. Alkaline phosphatase (ALP) staining at day 7 (**e**) and Alizarin Red S (ARS) staining at day 21 (**f**) post-treatment. **g** Protein expression and quantitative analysis of odontoblast differentiation-related markers in DPSCs after 7-day treatment. (Data are presented as the mean of >3 biological replicates ± SD. ^****^P < 0.000 1)

Considering the potential role of non-canonical Wnt ligands (Wnt5a) and canonical Wnt ligands (Wnt3a and Wnt10a) in inducing odontoblast differentiation,^17,18^ we analyzed the single-cell transcriptomic profiles of DPSCs treated with these three Wnt ligands. Single-cell transcriptome sequencing was performed on the four groups of samples 7 days after odontogenic induction. Following standard quality control parameters, we implemented a stringent filtering process to retain only high-quality cells for further analysis (Fig. S1a–f). After integration of four samples, we found that the Wnt3a group exhibited significant changes in Uniform Manifold Approximation and Projection (UMAP) distribution (Fig. 1a). Sample correlation analysis indicated strong correlations between the control, Wnt5a, and Wnt10a groups, while the Wnt3a group showed weaker correlations with the others (Fig. 1b). Analysis of differentially expressed genes demonstrated that Wnt3a treatment induced substantial transcriptional changes, including the activation of the Wnt downstream transcription factor *TCF7* and Wnt pathway antagonists *NKD1* and *WIF1*. In comparison, Wnt5a and Wnt10a groups exhibited relatively modest alterations in their gene expression profiles (Fig. 1c).

To better understand the effects of different Wnt ligands treatment, we performed GO enrichment analysis on the upregulated genes of each group compared with control group. The upregulated genes of Wnt3a group were associated with key biological processes such as the Wnt signaling pathway, extracellular matrix organization, mesenchymal cell differentiation, and odontogenesis (Fig. 1d). In contrast, the upregulated genes of the Wnt5a and Wnt10a groups did not identify biological processes related to odontogenic differentiation (Fig. S1g, h). Additionally, alkaline phosphatase (ALP) staining and Alizarin Red S (ARS) staining were performed on DPSCs treated for 7 and 21 days. The results showed significantly enhanced ALP activity and calcium nodule formation in the Wnt3a group (Fig. 1e, f). While the Wnt5a and Wnt10a groups showed slight increases in ALP staining compared with the control group, and no significant differences were observed in ARS staining. Furthermore, the expression levels of odontoblast differentiation-related proteins (such as DSPP and DMP1) were significantly increased in the Wnt3a-treated group (Fig. 1g). These results collectively indicate that Wnt3a significantly induces DPSC differentiation into mature odontoblasts, while Wnt5a and Wnt10a show relatively weaker induction effects.

### Wnt3a induces DPSCs to form an NKD1^+^ subpopulation with odontogenic differentiation function

**Fig. 2:**
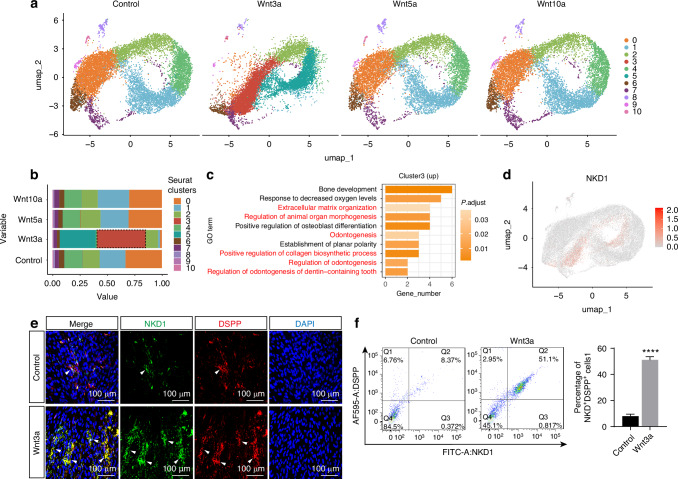
Wnt3a induces formation of an NKD1+ subpopulation with odontoblastic differentiation potential in DPSCs. **a** Cluster-resolved UMAP of scRNA-seq data from DPSCs treated with blank, Wnt3a, Wnt5a, and Wnt10a under odontogenic conditions. **b** Bar plot showing the proportional distribution of identified clusters. **c** GO enrichment analysis of upregulated genes of cluster 3. **d** FeaturePlot visualization of NKD1 expression pattern. **e** Immunofluorescence analysis of NKD1 co-localization with DSPP. White arrowheads indicate cells positive for both NKD1 and DSPP. **f** Flow cytometry quantification of NKD1^+^DSPP^+^ cell proportions. (Data are presented as the mean of >3 biological replicates ± SD. ^****^P < 0.000 1)

Overall, we identified 11 clusters from the four samples (Fig. 2a). Dotplot showed the marker genes of each cluster (Figs. 2a and S2a). Statistical analysis of cluster proportions revealed that a unique cluster 3, representing 44% of the Wnt3a-induced cells, was significantly induced (Fig. 2b). GO enrichment analysis of the upregulated genes in cluster 3 highlighted processes such as extracellular matrix organization and odontogenesis, suggesting that this cluster possesses odontogenic functionality (Fig. 2c). This cluster specifically highly expressed NKD1, therefore we named them the NKD1^+^ subpopulation (Fig. 2d). Besides, the NKD1^+^ subpopulation highly expressed several odontoblast differentiation-related genes, such as *COL1A1, COL1A2*, and *BMP4* (Fig. S2b), and Wnt receptors and target gene, such as *FZD2* and *TCF7*, suggesting that this subpopulation may respond strongly to canonical Wnt signaling (Fig. S2c). The pseudotime trajectory analysis revealed that the control group exhibited two branching trajectories, indicating multiple cell fate decisions under conventional odontogenic induction conditions (Fig. S2d, d’). In contrast, the Wnt3a group displayed a single linear trajectory, with the NKD1^+^ subpopulation located at the terminus of the trajectory, representing the more stringent differentiation pathway (Fig. S2e, e’).

We further validated the formation of the NKD1^+^ subpopulation in vitro using immunofluorescence staining. Multiplex immunofluorescence results demonstrated that this subpopulation was markedly enriched in the Wnt3a group, exhibiting robust expression of DSPP (Fig. 2e) and DMP1 (Fig. S2f). In contrast, only a minimal number of NKD1^+^ cells were detected in the control group, accompanied by noticeably lower expression levels of DSPP and DMP1. Flow cytometric analysis confirmed the presence and proportion of this subpopulation, revealing that 51.13% ± 2.45% of cells in the Wnt3a group were NKD1^+^ and DSPP^+^, significantly higher than the 7.92% ± 1.54% observed in the control group (Fig. 2f). Similar trends were observed in flow cytometric co-labeling experiments for NKD1 and DMP1 (Fig. S2g).

Given that clinical pulp regeneration treatments are closely related to inflammatory responses, it is of critical significance to deeply investigate the capacity of Wnt3a to induce NKD1^+^ cells and their functional state in an inflammatory pulpal microenvironment. We simulated the inflammatory environment by pre-treating DPSCs with 1 μg/mL lipopolysaccharide (LPS) for 24 h.^19^ Notably, even under inflammatory conditions, Wnt3a could still stably induce the differentiation of NKD1^+^ cells and significantly upregulate the expression levels of DSPP (Fig. S3a–d). Considering the potential advantages of Wnt small molecule agonists in clinical translation, we further evaluated two small molecule agonists, BIO and AZD2858.^20,21^ Our research found that the both agonists could mimic induction effects of Wnt3a, effectively promoting the differentiation of NKD1^+^ cells and maintaining the high expression characteristics of DSPP (Fig. S4a–d). These findings collectively reveal that canonical Wnt signaling pathway activation can robustly induce the differentiation of DPSCs into an NKD1^+^ subpopulation with odontogenic functionality.

### The NKD1^+^ subpopulation in the dentin-pulp complex represents secretory odontoblasts

**Fig. 3:**
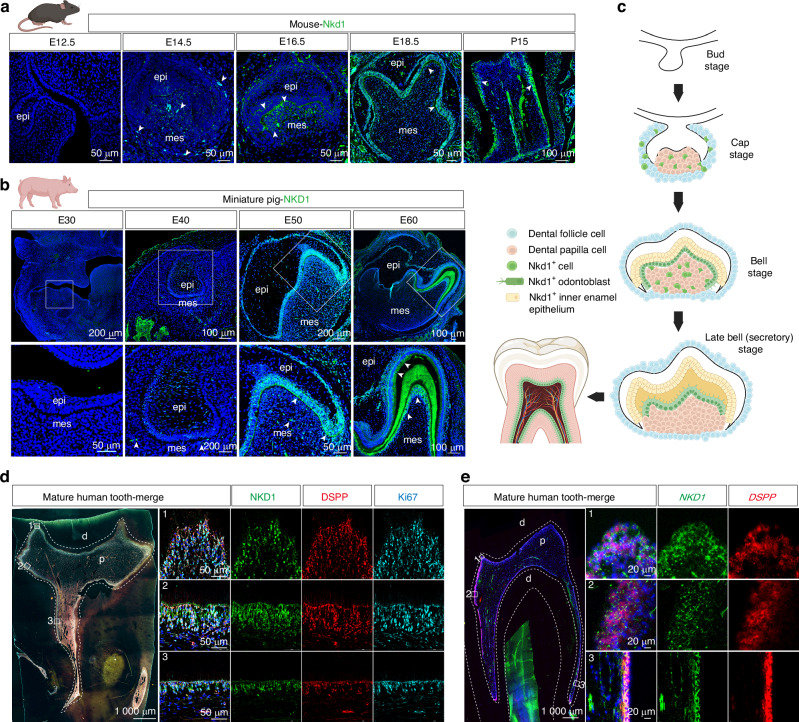
The NKD1+ subpopulation in the dentin-pulp complex represents secretory odontoblasts. Nkd1 protein expression patterns in tooth germs of mouse (**a**) and miniature pig (**b**) at sequential developmental stages. **c** Schematic representation of spatiotemporal Nkd1 expression during odontogenesis. **d** In fully developed human molars, immunofluorescence reveals NKD1 co-localization with DSPP^+^Ki67^+^ odontoblasts in the functional secretory layer. **e** In situ hybridization confirms coordinated *NKD1* and *DSPP* expression in mature odontoblast layers. (epi, epithelium; mes, mesenchyme; d dentin; p pulp.)

Mice and miniature pigs are the most commonly used model animals for studying tooth germ development. Among these, miniature pigs are considered an ideal model for dental development research due to their developmental pace, dentition type, and tooth structure, which closely resemble those of humans.^22^ We aim to investigate whether the NKD1^+^ subpopulation exists during tooth development. Immunofluorescence staining of tooth germs at various stages showed no Nkd1 signal in the bud stage of mouse (E12.5) and miniature pig (E30) tooth germs. Scattered Nkd1^+^ cells appeared in the cap stage of mouse (E14.5) and miniature pig (E40) tooth germs, localized in the dental follicle and mesenchyme. In the early bell stage of mouse (E16.5) and miniature pig (E50) tooth germs, prominent Nkd1^+^ cells were observed in the inner enamel epithelium and adjacent dental papilla. In the late bell stage of mouse (E18.5) and miniature pig (E60), Nkd1 signals persisted in the inner enamel epithelium, while in the dental papilla, signals became specialized in the odontoblast layer. Postnatally, mature odontoblasts in mice continued to express Nkd1 (Fig. 3a, b). The dynamic expression profile of Nkd1 during tooth development in mice and miniature pigs was similar to previously reported Wnt target genes (*β-Catenin, Axin2, Lef1*) and ultimately exhibited specific expression in the odontoblast layer,^23^ suggesting that the Nkd1^+^ odontoblast differentiation subpopulation might be the result of precise regulation of odontoblast differentiation by Wnt signaling (Fig. 3c).

Multiplex immunofluorescence mapping of human molar sections demonstrated conserved spatial distribution pattern of the NKD1^+^ subpopulation within odontoblast layers across distinct anatomical regions (cusp/cervical/root) in mature teeth. Strikingly, these cells demonstrated concurrent expression of three characteristic markers: DSPP, Ki67, and Nestin (Figs. 3d and S5), indicating their active dentin-secretory capacity and proliferative potential. In situ hybridization further confirmed spatial concordance between *NKD1* and *DSPP* mRNA signals along the odontoblast layer (Fig. 3e). Extending these observations to immature molars, we identified NKD1^+^ odontoblasts within cusp and cervical regions, which maintained robust DSPP/Ki67/Nestin co-expression (Fig. S6a, b). Given that root apex closure requires progressive differentiation of dental papilla progenitors into dentin-secreting odontoblasts,^24^ we specifically analyzed this dynamic region. Intriguingly, NKD1^+^ cells populated the non-closed apex microenvironment, displaying nuclear co-localization of NKD1 with Ki67 (Fig. S6a), suggesting their dual progenitor-proliferative identity. In situ hybridization demonstrated the co-accumulation of *NKD1-DSPP* mRNA along the apical sidewall (Fig. S6c), positioning NKD1 as both a functional marker and potential signaling orchestrator during progenitor-to-odontoblast transition. The persistent spatiotemporal coupling of NKD1^+^ cells with active dentinogenic niches, combined with their hybrid proliferative-secretory molecular signature, establishes this subpopulation as a mechanistic linchpin driving apex closure through accelerated dentin deposition.

### The NKD1-MSX1 axis promotes the odontogenic function of NKD1^+^ subpopulation

**Fig. 4:**
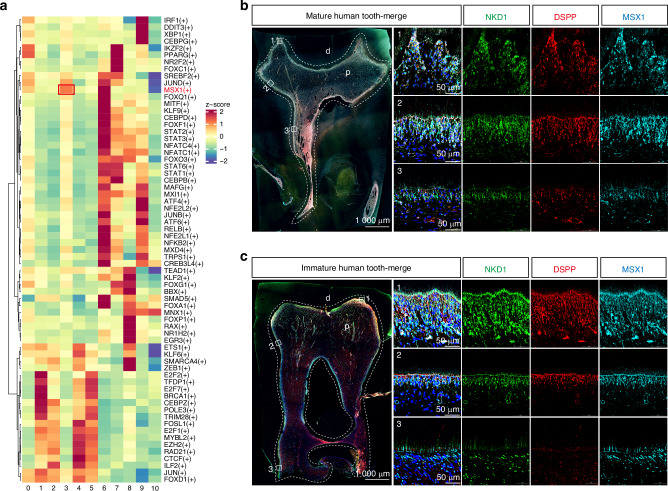
MSX1 is a transcription factor specifically activated in NKD1+ cells. **a** SCENIC analysis identifies MSX1 as the predominant activated transcription factor in the NKD1^+^ subpopulation of Wnt3a-treated samples. Immunofluorescence of fully developed (**b**) and developing (**c**) human dental samples demonstrates NKD1^+^ cell enrichment of MSX1 protein. d, dentin; p, pulp; ns, no significant difference. Data are presented as the mean of >3 biological replicates ± SD

To elucidate the mechanisms underlying the formation and odontogenic functionality of the NKD1^+^ subpopulation, we employed single-cell regulatory network inference and clustering (SCENIC) analysis to infer gene regulatory networks and identify key transcription factors mediating subpopulation formation. Notably, MSX1 emerged as the most highly activated transcription factor in the NKD1^+^ subpopulation (Figs. 4a and S7a), suggesting its pivotal role in the odontogenic function. This finding was further corroborated by immunofluorescence of human molar sections, which revealed specific MSX1 expression within the NKD1^+^ subpopulation (Fig. 4b, c). Intriguingly, while NKD1 and MSX1 were predominantly localized in the cytoplasm of odontoblasts in the tooth apex, they exhibited nuclear colocalization in the non-closed root apex region, with a high correlation coefficient indicating strong spatial overlap (Fig. S7b). STRING analysis (http://string-db.org/) identified potential physical and functional interactions between NKD1 and MSX1 (Fig. S7c). Given that impaired nuclear translocation of MSX1 is a known cause of tooth agenesis,^25^ we hypothesized that NKD1-mediated nuclear translocation of MSX1 might be a critical mechanism driving progenitor cell differentiation into odontoblasts.

**Fig. 5:**
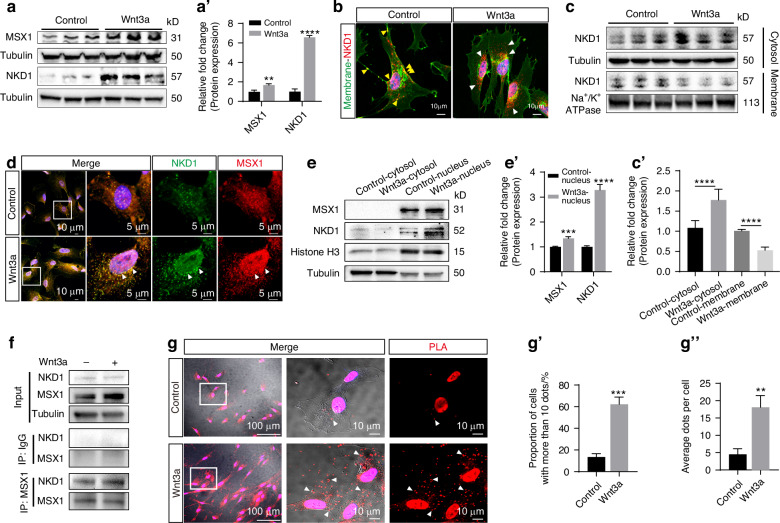
The NKD1-MSX1 axis promotes the odontogenic function of NKD1+ subpopulation. **a** Protein expression profiles and quantification of NKD1 and MSX1 in DPSCs at day 3 post-treatment (**a**’). **b** Subcellular localization analysis reveals membrane-associated NKD1 (yellow arrowheads) in untreated cells versus cytoplasmic redistribution (white arrowheads) following Wnt3a induction. **c** Membrane-cytoplasmic fractionation confirms Wnt3a-mediated NKD1 translocation. **d** Post-induction immunofluorescence shows nuclear envelope accumulation of co-localized NKD1/MSX1 (white arrowheads). **e** Nuclear-cytoplasmic fractionation verifies Wnt3a-enhanced nuclear accumulation of NKD1 and MSX1. **f** Co-IP confirms physical interaction between MSX1 and NKD1. **g** PLA demonstrates increased NKD1-MSX1 interaction events in Wnt3a-treated groups. White arrowheads show dots. (Data are presented as the mean of >3 biological replicates ± SD. ^**^P < 0.01, ^***^P < 0.001, ^****^P < 0.000 1)

First, we confirmed that Wnt3a treatment upregulated total protein levels of NKD1 and MSX1 in DPSCs (Fig. 5a), consistent with previous reports demonstrating their upregulation under canonical Wnt activation.^26,27^ Since the canonical Wnt ligand Wnt8 induces NKD1 relocalization from the inner cell membrane to exert negative feedback on Wnt signaling,^27^ we speculated that Wnt3a might similarly alter NKD1 subcellular localization. Immunofluorescence analysis revealed that Wnt3a treatment triggered NKD1 detachment from the cell membrane (Fig. 5b), and Membrane-cytosol fractionation assays demonstrated increased cytoplasmic NKD1 and decreased membrane-associated NKD1 in Wnt3a-treated cells (Fig. 5c). Furthermore, immunofluorescence demonstrated robust colocalization of NKD1 and MSX1 in Wnt3a-treated cells, with signals concentrated around the nuclear membrane, suggesting enhanced nuclear translocation of MSX1. In contrast, NKD1 and MSX1 were uniformly distributed in control cells, with no significant colocalization or nuclear accumulation (Fig. 5d). Nuclear-Cytoplasm fractionation assays revealed increased nuclear levels of both NKD1 and MSX1 in Wnt3a-treated cells (Fig. 5e). To determine whether NKD1 directly facilitates MSX1 nuclear translocation, we performed CO-IP assays, which confirmed a physical interaction between NKD1 and MSX1 (Fig. 5f). PLA results demonstrated that the formation of fluorescent dots (representing <40 nm proximity) specifically confirmed direct physical interaction between NKD1 and MSX1 (Fig. 5g).^28^ Quantification revealed significantly higher average dot counts per cell and increased proportion of cells with >10 dots in Wnt3a-treated groups compared to controls (Fig. 5g′, g′′), providing conclusive evidence that Wnt3a induces direct binding between NKD1 and MSX1 and facilitates NKD1-mediated nuclear translocation of MSX1.

### Genome-wide profiling of MSX1 transcriptional regulation in odontogenic differentiation

**Fig. 6:**
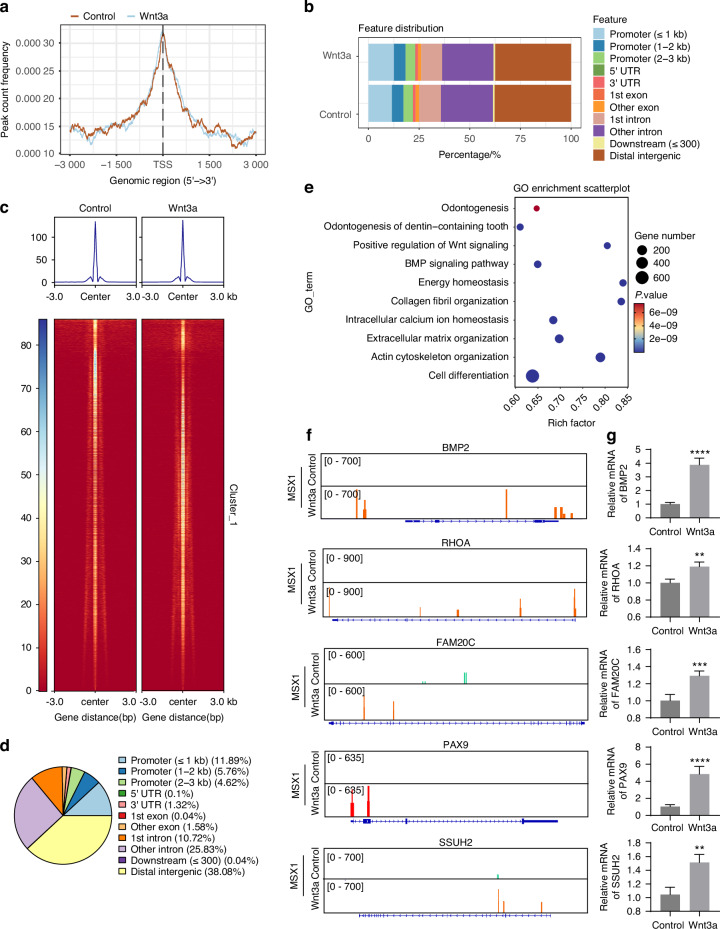
Genome-wide analysis of MSX1 transcriptional regulation in odontogenic differentiation. **a** Distribution of binding peaks relative to TSS. **b** Genomic annotation of peak distribution. **c** Heatmap showing differential peaks between Wnt3a treatment and control groups. **d** Genomic distribution of differential peaks between Wnt3a treatment and control groups. **e** GO enrichment analysis of differential peaks. **f** Peak profiles of genes related to tooth formation and dental development. **g** qRT-PCR validation of mRNA levels of genes involved in odontogenic differentiation. (Data are presented as the mean of >3 biological replicates ± SD. ^**^P < 0.01, ^***^P < 0.001, ^****^P < 0.000 1)

To validate MSX1 as the master transcriptional regulator of odontoblast differentiation, we performed CUT&Tag sequencing on DPSCs under Wnt3a stimulation. Peak calling analysis revealed comparable genomic distribution patterns of MSX1-binding peaks between Wnt3a-treated and control groups near transcription start sites (TSS) (Fig. 6a). However, Genomic annotation demonstrated preferential accumulation of Wnt3a-specific peaks in promoter regions (Fig. 6b). The heatmap of differential peaks demonstrates enhanced signal intensity in Wnt3a-treated groups (Fig. 6c). Notably, 22.25% of these differential peaks were mapped to promoter regions (Fig. 6d), accompanied by characteristic chromatin accessibility patterns at key odontogenic loci (Fig. 6f), collectively indicating Wnt3a-induced potentiation of MSX1-driven transcriptional regulation.

GO enrichment analysis of differential peaks revealed significant association with biological processes governing reparative dentinogenesis, including cell differentiation, extracellular matrix organization, Wnt/BMP signaling,^29^ and dentin development (Fig. 6e). Furthermore, the analysis revealed enrichment in pathways governing energy homeostasis and intracellular calcium ion dynamics, highlighting the crucial role of MSX1 in maintaining cellular homeostatic balance. These fundamental homeostatic mechanisms likely serve as key regulators in orchestrating odontogenic differentiation. Visualization of differential binding peaks highlighted MSX1 occupancy at promoters of functionally critical genes across multiple odontogenic pathways, including odontogenesis initiation (*PAX9*,^30^ *SSUH2*^31^), odontoblast differentiation commitment (*BMP2*,^32^ *RHOA*,^33^ *FAM20C*^34^) (Fig. 6f). The qRT-PCR results demonstrated that Wnt3a significantly upregulated the mRNA levels of *PAX9, SSUH2, BMP2, RHOA*, and *FAM20C* (Fig. 6g). These findings establish MSX1 as a direct transcriptional orchestrator that spatiotemporally coordinates odontogenic programs, mechanistically bridging Wnt3a signaling to functional dentin-pulp regeneration.

### NKD1^+^ DPSCs promote reparative dentin formation in pulp exposure models

To validate the in vivo reparative dentinogenic capacity of the NKD1^+^ subpopulation, we implemented a cell transplantation strategy in a pulp exposure model. Given the intracellular membrane localization of endogenous NKD1 protein (precluding FACS-based isolation), we engineered a NKD1-reporting lentivirus containing the NM_033119 promoter driving EGFP expression and puromycin resistance without altering endogenous NKD1 protein levels. After puromycin selection, the proportion of EGFP^+^ cells exceeded 95% (Fig. S8a), and the presence of the NKD1-reporting lentivirus did not affect NKD1 expression under Wnt3a treatment (Fig. S8b). NC^+^ DPSCs and NKD1^+^ DPSCs were prepared as single-cell suspensions for transplantation.

**Fig. 7:**
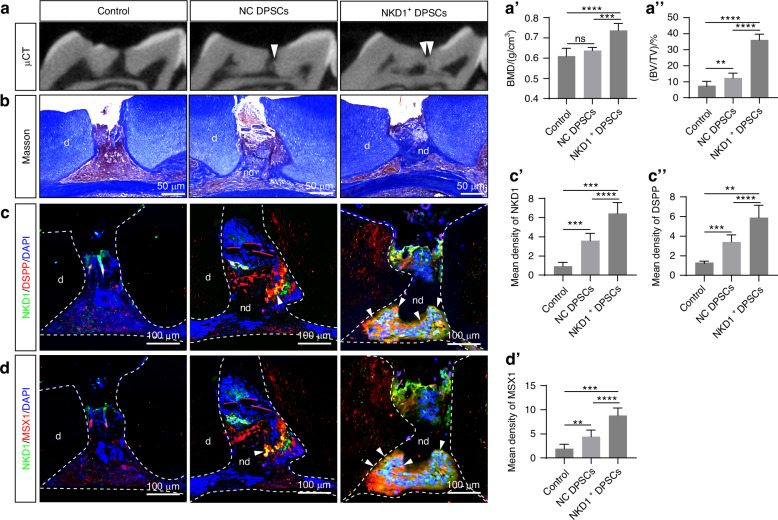
NKD1+ DPSCs promote reparative dentin formation in pulp exposure models. Analysis of reparative dentin formation in control group, NC DPSCs group, and NKD1^+^ DPSCs group at 6 weeks post-pulp exposure: **a** Representative μCT three-dimensional reconstruction images with corresponding BMD (**a**’) and BV/TV (**a**”) quantification; **b** Masson’s trichrome staining of the regenerated tissue at the pulp exposure site; Immunofluorescence analysis of the regenerated tissue showing co-staining of NKD1 with DSPP (**c**) and NKD1 with MSX1 (**d**), along with their respective mean fluorescence intensity quantification (**c**’, **c**”, **d**’), white arrowheads indicate cells positive for both NKD1 and DSPP (**c**) or NKD1 and MSX1 (**d**). (d, dentin; nd, newly formed dentin; ns, no significant difference. Data are presented as the mean of >3 biological replicates ± SD. **P < 0.01, ***P < 0.001, ****P < 0.000 1)

μCT 3D reconstruction at 6 weeks post transplantation revealed extensive dentin bridge-like hard tissue formation at the exposed pulp site in the NKD1^+^ DPSCs group, whereas NC DPSCs showed limited hard tissue deposition along lateral walls, and the cell-free control exhibited no significant regeneration (Fig. 7a). Quantitative bone microarchitecture analysis confirmed superior outcomes in the NKD1^+^ DPSCs group (BMD: (0.74 ± 0.03) g/cm³; BV/TV: 38.46% ± 5.02%) compared to NC DPSCs (BMD: (0.64 ± 0.01) g/cm³; BV/TV: 14.33% ± 4.17%) and controls (BMD: (0.61 ± 0.03) g/cm³; BV/TV: 4.82% ± 2.58%) (Fig. 7a’, a”).

Histological examination revealed similar patterns of reparative dentin formation consistent with the imaging results. Masson staining demonstrated no detectable mineralized tissue formation at the pulp exposure site in the control group. While the NC DPSCs group showed limited mineralization, characterized by sparse deposits of newly formed mineralized tissue along the margins of the exposure site, the NKD1^+^ DPSCs group displayed remarkable regenerative capacity. Specifically, NKD1^+^ DPSCs facilitated the formation of a complete dentin bridge that effectively sealed the pulp exposure site. The newly formed dentin bridge exhibited well-organized radial dentinal tubules. Notably, abundant vascular networks were observed underlying the dentin bridge, indicating robust pulpal vitality (Fig. 7b). Immunofluorescence staining at the pulp exposure site demonstrated intense NKD1, DSPP, and MSX1 fluorescent signals in the NKD1^+^ DPSCs group, whereas the NC DPSCs group exhibited relatively weaker signals, and the control group showed no significant positive staining (Fig. 7c, d’). These findings demonstrate that NKD1^+^ DPSCs possess the capacity to induce reparative dentin formation in vivo.

**Fig. 8:**
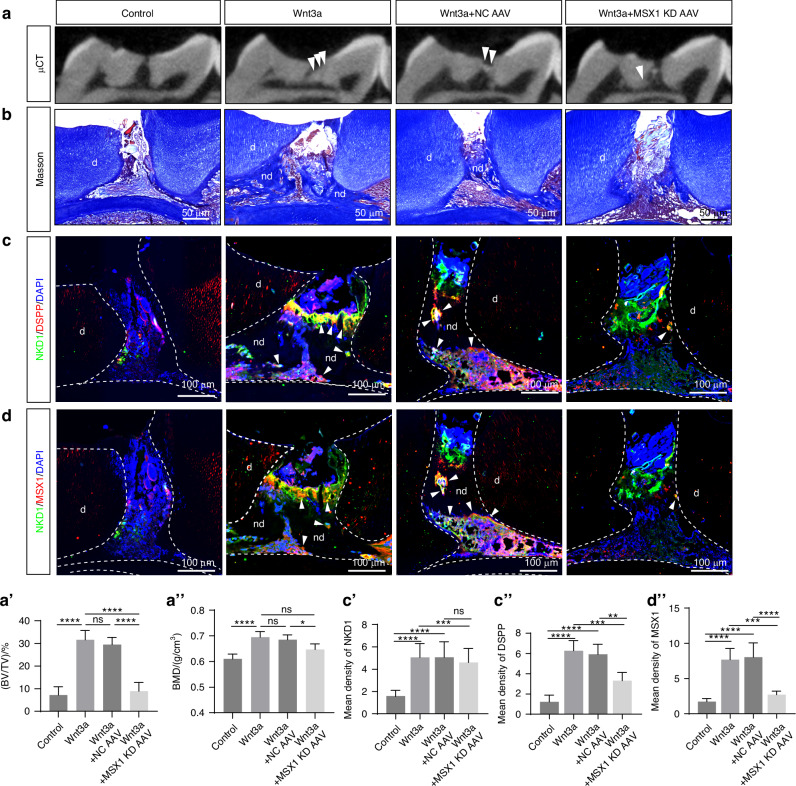
Wnt3a induces NKD1+ cells in vivo and promotes reparative dentinogenesis through the NKD1-MSX1 axis. Analysis of pulp repair in control group, Wnt3a group, Wnt3a+ NC AAV group, and Wnt3a+MSX1 KD AAV group at 6 weeks post-pulp exposure: **a** Representative μCT three-dimensional reconstruction images with corresponding BMD (**a**’) and BV/TV (**a**”) quantification; **b** Masson’s trichrome staining of the regenerated tissue at the pulp exposure site; Immunofluorescence analysis of the regenerated tissue showing co-staining of NKD1 with DSPP (**c**) and NKD1 with MSX1 (**d**), along with their respective mean fluorescence intensity quantification (**c**’, **c**”, **d**’), white arrowheads indicate cells positive for both NKD1 and DSPP (**c**) or NKD1 and MSX1 (**d**). (d, dentin; nd, newly formed dentin; ns, no significant difference. Data are presented as the mean of >3 biological replicates ± SD. ^*^P < 0.05, ^**^P < 0.01, ^***^P < 0.001, ^****^P < 0.000 1)

To further validate whether Wnt3a could induce the NKD1^+^ subpopulation in vivo, we established a cell-free regeneration strategy using the pulp exposure model, while assessing the role of NKD1-MSX1 axis in reparative dentinogenesis through adeno-associated virus (AAV)-mediated MSX1 knockdown (MSX1 KD AAV) in situ. Wnt3a treatment induced robust dentin bridge-like mineralization at exposure sites, whereas controls showed negligible hard tissue formation. Comparable therapeutic outcomes between Wnt3a+NC AAV and Wnt3a alone confirmed AAV biocompatibility. In contrast, Wnt3a+MSX1-KD AAV exhibited limited mineralization, demonstrating the indispensable role of NKD1-MSX1 axis (Fig. 8a). Quantitative micro-CT analysis revealed significantly higher bone mineral density (BMD) (Wnt3a: (0.69 ± 0.03) g/cm³; Wnt3a+NC AAV: (0.67 ± 0.02) g/cm³) and BV/TV (Wnt3a: 31.14% ± 3.55%; Wnt3a+NC AAV: 28.89% ± 2.81%) compared to Wnt3a+MSX1-KD AAV (BMD: (0.66 ± 0.02) g/cm³; BV/TV: 10.89% ± 4.74%), conclusively establishing this axis as an essential regulator of mineralization efficacy (Fig. 8a’, a”).

Masson staining analysis revealed that, compared to the control group, Wnt3a treatment significantly enhanced hard tissue formation at the pulp exposure site, characterized by extensive continuous mineralized tissue deposition with a tendency to form complete dentin bridge structures. This promotional effect was maintained in the Wnt3a+NC AAV group; however, following MSX1 knockdown (Wnt3a+MSX1-KD AAV group), the effect was substantially attenuated, with limited mineralized tissue formation confined to the defect margins (Fig. 8b). Further immunofluorescence analysis confirmed that local application of Wnt3a in vivo induced the generation of NKD1^+^ cells, which exhibited strong expression of DSPP and MSX1. Moreover, knockdown of MSX1 resulted in decreased expression of DSPP and MSX1 (Fig. 8c, d’). The above findings confirm that Wnt3a is capable of inducing NKD1^+^ cell formation in vivo, while also establishing that the NKD1-MSX1 pathway is indispensable for mature odontoblast differentiation.

## Discussion

**Fig. 9:**
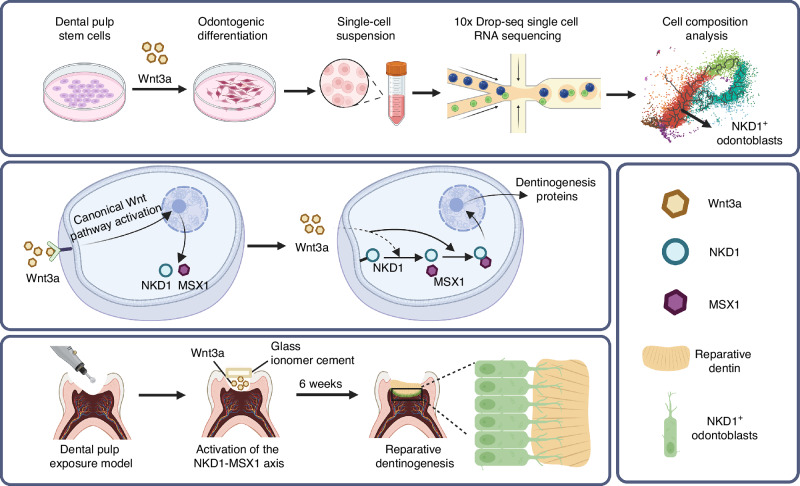
Schematic diagram illustrating the mechanism by which Wnt3a induces odontogenic differentiation of dental pulp stem cells via NKD1-MSX1 axis to promote in situ dentin formation

Dentin regeneration presents unique challenges distinct from bone repair, primarily due to its specialized microtubular structure and physiological demands. Current dentin regeneration studies exhibit significant variability in outcomes. Our research aims to identify a specific subpopulation of stem cells with a stronger potential for dentin regeneration to optimize dentin regeneration strategies. Through various techniques such as single-cell sequencing and CUT&Tag, we successfully identified a Wnt3a-inducible NKD1^+^ odontoblastic subpopulation with potent dentinogenic activity. This subpopulation was consistently observed across multiple species (mouse, miniature pig, and human) in both developing and mature odontoblast layers. Importantly, we established the NKD1-MSX1 signaling axis as a central regulatory mechanism, with pulp exposure models directly implicating NKD1^+^ cells in reparative dentin deposition (Fig. 9). These findings collectively advance our understanding of odontoblast lineage in dental regeneration.

Our single-cell transcriptome analysis systematically reveals substantial heterogeneity within DPSC populations, providing a cellular-level explanation for these clinical variations. This heterogeneity simultaneously presents both challenges and opportunities. The challenge lies in how we can effectively induce heterogeneous stem cells into a state suitable for tissue regeneration and improve the efficiency of tissue regeneration. The opportunity lies in that by identifying and enriching specific stem cell subpopulations with defined differentiation potential, we can significantly improve the predictability and effectiveness of dentin regeneration therapies. In this study, we found that the NKD1^+^ subpopulation does indeed have a better dentin regeneration effect in vivo. Through this discovery pathway, by elucidating key functional subpopulations and their formation mechanisms in tissue-specific regeneration—such as bone regeneration—the prospect of efficiently achieving comprehensive tissue reconstruction is progressively emerging.

After induction by Wnt3a, the expression of *NKD1* significantly increased, ranking first among the differentially expressed genes, suggesting that it is a direct target gene for the Wnt signaling response. Therefore, we take it as the key marker gene of the specific subpopulation cluster3 induced by Wnt3a. Notably, NKD1 exhibits dual functionality beyond its role as a subpopulation marker. Analogous to Axin2 in canonical Wnt signaling,^27^ NKD1 operates as both a negative feedback regulator and activation marker. This duality is exemplified by NKD1-mediated Wnt inhibition and the jaw hypoplasia shared between NKD1^−/−^ and Axin2^−/−^ mice.^35,36^ Intriguingly, Wnt3a stimulation triggers NKD1 relocalization from the membrane to cytoplasmic/nuclear domains, a spatial shift that facilitates MSX1 nuclear translocation—a prerequisite for odontogenic activation. This mechanism underscores the contextual plasticity of Wnt signaling components in tissue-specific regeneration.

The spatiotemporal regulation of MSX1 emerges as a linchpin in odontoblast differentiation.^30,37^ While the essential role of MSX1 in tooth development is well-documented,^38–40^ our work reveals its extended functionality in adult stem cell differentiation. Canonical Wnt signaling stabilizes MSX1 transcriptional activity.^26^ Embryonically, MSX1 potentiates Wnt signaling via Dkk2/Sfrp2 suppression^41,42^ and synergizes with PAX9 to amplify BMP4 transactivation.^43,44^ Our CUT&Tag analysis reveals its adult-specific role in DPSC differentiation. Wnt3a induces MSX1 binding at promoters of odontogenic regulators (*PAX9, SSUH2*) and mineralization effectors (*BMP2, RHOA, FAM20C*), suggesting developmental stage-specific regulatory modules. Furthermore, MSX1 co-activates energy and calcium ion-homeostasis genes, unveiling previously unrecognized pathways in lineage stabilization.

Several limitations guide future research directions. First, the dynamic behavior of NKD1^+^ cells during development and repair remains uncharacterized—a gap addressable through NKD1-CreERT2 lineage-tracing models complemented by computational analysis of public single-cell datasets. Second, while our in vitro evidence strongly supports the chaperone function of NKD1 for MSX1, conditional knockout models are needed to confirm its in vivo necessity. Third, although complete dentin bridge formation was observed at pulp exposure sites in the NKD1^+^ DPSC transplantation group, it remains unclear whether the newly formed tissue was directly generated by transplanted NKD1^+^ DPSCs or resulted from host odontoblast differentiation induced by the regenerative microenvironment created by NKD1^+^ DPSCs. This suggests that while investigating the intrinsic functions of NKD1^+^ DPSCs, we should also explore their secretome profile to better understand their regenerative capabilities.

In conclusion, this work pioneers the identification of a therapeutically relevant NKD1^+^ odontoblast subpopulation and delineates its regulatory axis. The discovery of NKD1-mediated MSX1 nuclear translocation redefines our understanding of Wnt signaling in dental regeneration, bridging developmental biology and regenerative medicine. By elucidating conserved mechanisms, these findings provide a robust framework for developing targeted strategies in regenerative endodontic treatment.

## Materials and methods

### Cell culture

Human DPSCs were purchased from Procell Co. Cells were maintained in α-MEM (Gibco) supplemented with 10% FBS (Gibco), 100 μg/mL penicillin, and 100 mg/mL streptomycin (Gibco) at 37 °C with 5% CO₂. Cells were cryopreserved by resuspending in serum-free cell cryopreservation medium, transferred into cryovials (NEST Biotechnology), and stored in liquid nitrogen.

For odontoblastic induction, cells were cultured in odontoblastic differentiation medium (ODM: complete growth medium with 50 mg/L ascorbic acid, 10 mmol/L β-glycerophosphate, and 100 nmol/L dexamethasone). Experimental groups received ODM supplemented with 50 ng/mL recombinant human Wnt3a (R&D Systems, 5036-WN-010),^10^ Wnt5a (Abcam, ab204627), or Wnt10a (Abcam, ab289784). DPSCs were pretreated with 1 μg/mL E. coli lipopolysaccharide (LPS, Solarbio) for 24 h to induce an inflammatory state. To explore the induction effects on NKD1^+^ cells, 50 nmol/L Wnt small molecule agonists BIO (Selleck) and AZD2858 (MCE) were used.

### Sample preparation & single-cell RNA sequencing (scRNA-seq)

Cells at ~40%–50% confluency were treated with Wnt ligand-supplemented ODM containing 50 ng/mL of recombinant human Wnt3a, Wnt5a, or Wnt10a for 7 days (culture medium was refreshed every 48 h). Single-cell suspensions were prepared using enzymatic dissociation, with cell viability >85% confirmed by Countstar Automated Cell Counter. All the scRNA-seq libraries were prepared on 10x Genomics Chromium System (Single Cell 3’ Regent Kits V3) and sequenced (Illumina NovaSeq 6000, 150 bp paired-end) by Jinkerui Medical Laboratory (Beijing).

### Data processing

Raw FASTQ files were aligned to the GRCh38 human reference genome (https://cf.10xgenomics.com/supp/spatial-exp/refdata-gex-GRCh38-2020-A.tar.gz) using DNBC4tools (MGI Tech Co., Ltd, China) to obtain the raw count matrices. The relative statistics were showed in Table 1. All 4 samples were integrated and analyzed following the standard pipeline of the Seurat package (v3.2). We only keep good quality cells that meet the following criteria: (1) cells with between 1 000 and 9 000 genes expressed; (2) cells with UMI counts between 600 and 80 000; (3) cells with mitochondrial genes expression percentages fewer than 10%; (4) cells with hemoglobin genes expression percentages fewer than 25%. Final cell numbers per group were: Control (11 363), Wnt3a (10 938), Wnt5a (8 875), Wnt10a (9 149).

**Table 1:** Statistics related to the single-cell raw count matrices

Sample name	Control	Wnt3a	Wnt5a	Wnt10a
Species	Human	Human	Human	Human
Estimated number of cell	11 686	11 086	9 198	9 408
Mean reads per cell	34 300	31 556	44 067	45 733
Mean UMI count per cell	16 656	17 310	21 081	20 873
Median UMI counts per cell	12 441	13 464	15 467	15 160
Total genes detected	33 215	34 054	34 286	34 460
Mean genes per cell	4 397	4 517	4 945	4 973
Median genes per cell	4 150	4 304	4 725	4 716
Sequencing saturation	44.87%	37.89%	45.62%	48.25%
Fraction Reads in cell	80.87%	83.91%	80.42%	79.80%

### Dimensionality reduction, cell clustering and identification

The filtered gene expression matrix was normalized and log-transformed. Highly variable genes were calculated and used to perform principal component analysis. Louvain clustering was performed on all cells with the ‘FindClusters’ function using the first 50 PCs and a resolution of 0.6. UMAP was used to visualize single-cell clusters in a reduced 2D space. Differential gene expression was calculated using the “FindAllMarkers” function (settings: min.pct = 0.25, thresh.use = 0.25). Gene Ontology (GO) characteristics of gene clusters were determined using the clusterProfiler package (version 3.8.1) with default parameters.

### Trajectory inference

The pseudo-temporal trajectory was reconstructed with Monocle2 (version 2.14.0) and Monocle3 (version 1.3.7). For Monocle2, we followed the official vignette with recommended parameters. First, 3 000 highly variable genes defined in Seurat were identified as ordering genes for ordering cells. Then, the Discriminative Dimensionality Reduction with Trees method was used to construct development path. The monocle3 analysis was conducted using default parameters, with the proliferating cell population selected as the starting root node for pseudo-temporal trajectory differentiation.

### Transcription factor (TF) score analysis

The SCENIC pipeline (pySCENIC version 1.3.1) was used to predict transcription factors and putative target genes regulated throughout odontogenic differentiation. First, gene regulatory interactions were calculated based on co-expression across the single cell dataset with GRNBoost2, followed by pruning interactions using known TF binding motifs and the construction of dataset-specific regulatory modules (regulons). Regulons were then scored in each individual cell using AUCell.

### Alkaline phosphatase (ALP) and Alizarin Red S (ARS) staining

To assess odontoblastic differentiation, ALP activity and calcium deposition were analyzed at predetermined time points following odontogenic induction. Alkaline Phosphatase Assay Kit (Beyotime) and Alizarin Red S solution (Cyagen) were used according to the manufacturer’s protocol.

### Western blotting

Total cellular protein was extracted using RIPA lysis buffer (Beyotime) supplemented with a protease inhibitor cocktail (Beyotime) and 1 mM PMSF. For cell membrane and cytoplasmic protein separation, a cell membrane protein and cytoplasmic protein extraction kit (Beyotime) was used. Nuclear and cytoplasmic proteins were extracted using NE-PER™ Nuclear and Cytoplasmic Extraction Reagents (Thermo Scientific), following the manufacturer’s instructions. Tri-color prestained protein marker (Shandong Sparkjade Biotechnology Co., Ltd) was used to determine protein molecular weights. Protein samples (10 μg) were separated on 4%–20% SurePAGE™ precast gels (GenScript) and transferred onto PVDF membranes (Millipore). Membranes were blocked with QuickBlock™ Western blocking buffer (Beyotime) at room temperature for 15 min, followed by overnight incubation at 4 °C with primary antibodies specific for DSPP (sc-73632, 1:200, Santa Cruz), DMP-1 (sc-73633, 1:200, Santa Cruz), MSX1 (NBP2-24715SS, 1:300, NOVUS), NKD1 (orb577992, 1:1 000, Biorbyt), beta-Tubulin (EM0103, 1:5 000, HUABIO), Na^+^/K^+^-ATPase (A11683, 1:50 000, ABclonal), and Histone H3 (17168-1-AP, 1:2 000, Proteintech). Membranes were then incubated with HRP-conjugated secondary antibodies (Goat anti-Rabbit IgG, HA1001, 1:50 000, HUABIO; Goat anti-Mouse IgG, HA1006, 1:20 000, HUABIO) for 1 h and visualized using an ECL chemiluminescence kit (Beyotime).

### Tissue preparation

All animal experimental procedures were approved by the Animal Ethics Committee of Capital Medical University (approval number: AEEI-2022-185). Tooth germ samples were collected from C57BL/6J mice and Chinese miniature pigs. Mouse first molars (M1) were harvested at embryonic days 12.5 (E12.5, bud stage), 14.5 (E14.5, cap stage), 16.5 (E16.5, early bell stage), and 18.5 (E18.5, late bell stage). Third deciduous molars (DM3) from miniature pigs were collected at E30 (bud stage), E40 (cap stage), E50 (early bell stage), and E60 (late bell stage). Human third molars tissue samples were generously provided by Professor Jianguang Xu.

Samples were fixed in 4% paraformaldehyde (PFA) at 4 °C for 24 h. Decalcification was conducted in 10% ethylenediaminetetraacetic acid (EDTA, pH 7.4), with mouse and miniature pig samples undergoing 7–14 days of treatment, and human samples undergoing 60 days of treatment. Following dehydration through an ethanol gradient series, tissues were paraffin-embedded. Coronal sections (4 μm thickness) were prepared for mouse and pig samples, while sagittal sections (4 μm thickness) were obtained for human samples.

### Immunofluorescence

For cell membrane visualization, cultured cells were labeled with Alexa Fluor 488-conjugated Wheat Germ Agglutinin (WGA; 1:200, Thermo Fisher Scientific) prior to fixation. Immunofluorescence of tissue samples was performed as described previously.^45^ Briefly, deparaffinized sections underwent antigen retrieval and were treated with 3% H₂O₂ in methanol for 15 min to quench endogenous peroxidase activity. Sections were then blocked with goat serum for 1 h at room temperature. Primary antibodies were applied overnight at 4 °C, including DSPP (sc-73632, 1:50, Santa Cruz), DMP-1 (sc-73633, 1:50, Santa Cruz), MSX1 (NBP2-24715SS, 1:200, NOVUS), NKD1 (orb577992, 1:50, Biorbyt), Ki67 (ab15580, 1:200, Abcam), and Nestin (A11861, 1:100, ABclonal). Secondary antibodies, including FITC-conjugated (HA1004, HA1003, 1:500, HUABIO), iFluor™ 594-conjugated (HA1122, HA1126, 1:500, HUABIO) and iFluor™ 647-conjugated antibodies (HA1123, HA1127, 1:500, HUABIO), were applied for 1 h. Sections were mounted using Fluoroshield™ mounting medium with DAPI (Sigma) and imaged using a Nikon microscope. Fluorescence signal colocalization was analyzed using NIS-Elements imaging software. The mean fluorescence intensity was quantified using ImageJ software.

### Flow cytometry

Flow cytometric analysis was performed using the same antibody staining protocols as described for immunofluorescence. Primary antibodies include DSPP (sc-73632, 1:50, Santa Cruz), DMP-1 (sc-73633, 1:50, Santa Cruz), and NKD1 (orb577992, 1:50, Biorbyt). Secondary antibodies include FITC-conjugated (HA1004, 1:500, HUABIO) and iFluor™ 594-conjugated (HA1126, 1:500, HUABIO). Unstained samples were used as negative controls to establish proper gating strategies. The sort gating strategy incorporated live cell and singlet gates prior to gating on individual markers. Samples were analyzed on a BD LSRFortessa flow cytometer (BD Biosciences) and data were processed using FlowJo software.

### In situ hybridization

Fresh third molars were fixed in specialized animal in situ hybridization fixative overnight at 4 °C. The samples were decalcified, dehydrated, and embedded in paraffin. Sections of 5 μm thickness were prepared for in situ hybridization.

The in situ hybridization experiments were conducted by Servicebio. Briefly, after deparaffinization and antigen retrieval, sections were treated with Proteinase K (20 μg/mL) at 40 °C to enhance probe penetration. Pre-hybridization was performed at 40 °C for 1 h to ensure optimal probe binding. This was followed by overnight hybridization with primary probes at 40 °C. For signal amplification, the sections underwent branch probe hybridization at 40 °C for 45 min and signal probe hybridization at a 1:200 dilution for 3 h at 40 °C. Stringent washing steps were performed using a gradient of SSC buffers (2×SSC to 0.1×SSC) at 40 °C to minimize nonspecific binding. Nuclei were counterstained with DAPI for 8 min in the dark before mounting with antifade medium to preserve fluorescence. Fluorescent imaging was conducted using a Nikon upright microscope equipped with standardized filters for detection. Detailed information regarding the probes used in this study is provided in Table 2.

**Table 2:** Information on in situ hybridization probes

Serial number	Name	Sequence (5’ to 3’)	Modification
1	DSPP	CATGCACCAGGACACCACTTTCTTTGCATCGGAATTATCCAGGCCAGCATCTTTGGCTCTTCCCAACAGCATGTGTCTTCTCCTCGGCTACTGCTGTTATTGTCGTGGTTACTGTCACTGCCTTCACTGT	
	Branch Probe H2		
	Signal Probe H2		Cyanine3
2	NKD1-H3	GTGACCTTGCCGTTGTTGTCAAAGTCCTTTACCCGCAGCATCTTGCTGGATGTTTCTCCTCTCGATGTTCTCATCTACGCGTTGCTGGAGCTCTGAGACCTTGG	
	Branch Probe H3		
	Signal Probe H3		Alexa Fluor 488

### Co-immunoprecipitation (CO-IP)

For CO-IP, cells were harvested and lysed for 20 min in lysis buffer containing 20 mmol/L Tris-HCl (pH 7.4), 150 mmol/L NaCl, 1% Triton X-100, 1 mmol/L PMSF, and protease inhibitor cocktail. A total of 2 mg of cell lysates were immunoprecipitated with 6 μL of MSX1 antibody (H00004487-M11, Novus) or IgG control (sc-515946, Santa Cruz) at 4 °C overnight. Protein-antibody complexes were captured by incubation with 50 μL of Dynabeads™ Protein G (Invitrogen) at 4 °C for 2 h. Beads were washed with lysis buffer, and bound proteins were eluted in 4X LDS Sample Buffer (GenScript) by boiling at 70 °C for 10 min. Samples were seperated on 4–20% SurePAGE™ precast protein gels (GenScript) and analyzed by Western blotting.

### Proximity ligation assay (PLA)

After seeding DPSCs on chamber slides and completing induction, cells were fixed and permeabilized for pre-treatment. Samples were blocked with Duolink® blocking solution (Sigma-Aldrich) at 37 °C for 60 min. Primary antibodies, MSX1 (H00004487-M11, 1:100, Novus) and NKD1 (orb577992, 1:50, Biorbyt), were diluted and incubated with the samples at room temperature for 2 h. After washing with Duolink® PLA wash buffer (Sigma-Aldrich), samples were incubated with Duolink® PLA probes (anti-rabbit PLUS and anti-mouse MINUS; Sigma-Aldrich) at 37 °C for 1 h. Following additional washes, ligation was performed by incubating with ligase at 37 °C for 30 min. Amplification was achieved by incubating with polymerase at 37 °C for 100 min. After final washes, samples were mounted with Duolink® mounting medium and imaged using a Nikon fluorescence microscope. The average number of PLA dots per cell and the proportion of cells with more than 10 dots per field of view was calculated for quantitative analysis.

### CUT&Tag (cleavage under targets and tagmentation)

Prior to CUT&Tag processing, DPSCs underwent rigorous mycoplasma decontamination using Anti-Mycoplasma Elimination Reagent (Procell), with clearance confirmed via MycAway™ Plus-Color One-Step Mycoplasma Detection Kit (Yeasen). Cells were initially conjugated with Concanavalin A-coated magnetic beads to facilitate surface glycoprotein-mediated immobilization. Following permeabilization with digitonin, MSX1 antibodies were introduced, enabling intracellular penetration and antigen binding. Hyperactive pG-Tn5 or pA-Tn5 Transposase (selected based on antibody compatibility) was then incubated with the cells, wherein the Protein G/A domain directed Tn5 enzyme localization to antibody-bound regions via Fc region interactions. Transposase activation in optimized buffer simultaneously fragmented chromatin proximal to target proteins and ligated Illumina sequencing adapters to DNA termini. Fragmented DNA was extracted via phenol-chloroform purification, followed by PCR amplification using adapter-specific primers to enrich libraries, which were subsequently purified and quality-controlled via Agilent 2100 Bioanalyzer and Qubit quantification. Sequencing was performed on the Illumina NovaSeq platform.

For bioinformatics analysis, raw reads underwent quality trimming (Trimmomatic) and alignment to the reference genome, with FastQC assessing data integrity. High-confidence mapped reads were subjected to peak calling using MACS2, followed by IDR-based peak filtering for reproducible signal identification. ChIPseeker annotated genomic features associated with peaks, while HOMER performed de novo motif analysis. Differential peak analysis across experimental groups was conducted via DiffBind.

### Quantitative real-time polymerase chain reaction (qRT-PCR)

Total RNA was extracted using the RNAeasy™ Animal RNA Isolation Kit (Beyotime). RNA concentration and purity were determined using a NanoDrop 2000 spectrophotometer (Thermo Fisher). cDNA synthesis was performed using the PrimeScript™ RT Reagent Kit (TaKaRa) according to the manufacturer’s instructions. qRT-PCR analysis was carried out using the TB Green® Premix Ex Taq™ II Kit (TaKaRa Bio) on a CFX96 Real-Time PCR Detection System. The thermal cycling conditions consisted of an initial denaturation at 95 °C for 30 s, followed by 40 cycles of 95 °C for 5 s, 60 °C for 30 s. Relative gene expression was calculated using the 2^−ΔΔCT^ method, with GAPDH as the internal reference gene. All primer sequences used in this study are listed in Table 3.

**Table 3:** Primer sequences

Gene	Forward sequence	Reverse sequence
BMP2	TTCGGCCTGAAACAGAGACC	CCTGAGTGCCTGCGATACAG
RHOA	AGCCTGTGGAAAGACATGCTT	TCAAACACTGTGGGCACATAC
PAX9	GGAGGAGTGTTCGTGAACGG	CGGCTGATGTCACACGGTC
SSUH2	AAGGTCCTCCGATGTTTCAGG	ATTTGTGGCATTCCTTGACCA
FAM20C	GGCACAATGCGGAGATTGC	CAGAGCTTCTTGTCCCGTGT

### Lentivirus construction and cell transfection

To establish NKD1-labeled DPSCs for transplantation, we developed a promoter-specific lentiviral system (GenChem Inc., KV208 backbone) containing NM_033119-promoter sequence without interfering endogenous NKD1 expression. The modified vector (NM_033119-promoter-EGFP-SV40-Puromycin) was packaged in 293T cells and concentrated via ultracentrifugation.

DPSCs were transduced with NKD1-EGFP lentivirus (MOI = 40), followed by 7 days of odontogenic induction with 50 ng/mL Wnt3a (R&D Systems) and 2 days of puromycin selection at 2 μg/mL to obtain NKD1^+^ DPSCs. Additionally, DPSCs were transduced with NC-EGFP lentivirus (MOI = 40) and then subjected to 7 days of odontogenic induction and 2 days of puromycin selection at 2 μg/mL to generate NC DPSCs. Both NC DPSCs and NKD1^+^ DPSCs were subsequently resuspended at a concentration of 5 × 10^4^ cells per μL in MEM for in vivo transplantation.

### Adeno-associated virus construction

The AAV for MSX1 Knockdown were synthesized by Cyagen Biosciences for local transfection in mouse dental pulp. The sequence of MSX1 Knockdown (KD) AAV: AAV9-U6>-CMV>Kozak-SaCas9-HA. The sequence of negative control (NC) AAV: AAV9-CMV>Kozak-EGFP.

### Pulp exposure and reparative dentin formation

Adult male nude mice (6 weeks old) were used for in vivo molar pulp exposure experiments. All experimental procedures were approved by the Capital Medical University Animal Care and Use Committee (IACUC Protocol Number: AEEI-2024-222).

Pulp exposure and subsequent drug administration were performed according to previously established protocols.^46^ Specifically, all surgical procedures were conducted under general anesthesia using 3% (w/v) pentobarbital sodium. Access to the dentin of maxillary molars was achieved using a round carbide burr (FG 1/4) mounted on a high-speed dental handpiece. Following dentin exposure, pulp chamber access was established using a 6G needle.

For cellular regeneration, 1 µL of NKD1^+^ single-cell suspension was impregnated into sterile collagen sponge scaffolds (0.1 mm^3^) and surgically positioned within pulp exposure sites. In cell-free regenerative approaches, identical collagen scaffolds were pre-loaded with either 0.1 µL PBS, 50 ng/mL Wnt3a (R&D Systems) or combinatorial treatment (50 ng/mL Wnt3a + NC AAV (1 × 10^8^ PFU), 50 ng/mL Wnt3a+MSX1 KD AAV (1 × 10^8^ PFU)). All interventions were secured with glass ionomer cement under surgical microscopy. Postoperative animals were maintained on soft feed for 7 days to minimize mechanical disturbance at the operative site.

6 weeks post surgery, mice were euthanized, and maxillae were harvested and fixed in 4% paraformaldehyde. Specimens were scanned using a Scanco µCT50 micro-computed tomography (µCT) scanner with the following parameters: 70 kV X-ray source and 7.5 µm resolution. The density of newly formed tissue was calculated using calibration phantoms provided by the µCT manufacturer. A standardized cylindrical region of interest (ROI: 0.06 mm diameter × 0.2 mm height) was aligned to the pulp exposure site for mineralization quantification. BMD and percent bone volume (BV/TV) were calculated to quantify the mineralization status of the regenerated tissue.

After µCT scanning, samples were decalcified, dehydrated, embedded in paraffin, and sectioned for histological analysis. Sections were stained with Masson’s trichrome and immunofluorescence staining for further evaluation.

### Statistical analysis

Quantitative data were expressed as mean ± standard deviation. Statistical analyzes were performed with Prism 10.0 software, employing Student’s *t*-test for paired comparisons and ANOVA for multiple comparisons. A significance level of *P* < 0.05 was applied to determine statistical significance.

## Supplementary information

Supplementary information

## Data Availability

All other relevant data supporting the key findings of this study are available within the article and its Supplementary Information files or from the corresponding author upon reasonable request.
