# Supplementary material for: Wnt3a promotes in situ dentin formation through NKD1-MSX1 axis-mediated odontogenic differentiation of dental pulp stem cells
Source: Int J Oral Sci. 2026 Jan 13;18:9. doi: 10.1038/s41368-025-00406-3 (PMC12796365; doi:10.1038/s41368-025-00406-3)
Supplement: Supplementary file 1 — Supplementary information [file 41368_2025_406_MOESM1_ESM.pdf]

Wnt3a Promotes in situ Dentin Formation through NKD1-MSX1 Axis-Mediated Odontogenic Differentiation of Dental Pulp Stem Cells

Haoran Du, Qiong Li, Chenchen Zhou, Junji Xu, Kang Gao, Zixiao Li, Yifan Xu, Ousheng Liu, Bing Li, Jianguang Xu, Jingsong Wang, Hideaki Kagami, Xianqi Li, Su Chen, Jian Zhou*

*Correspondence to: Zhoujian@ccmu.edu.cn (J. Z.)

This file includes:

Figures S1 to S8

This table includes:

Table 1 to 3

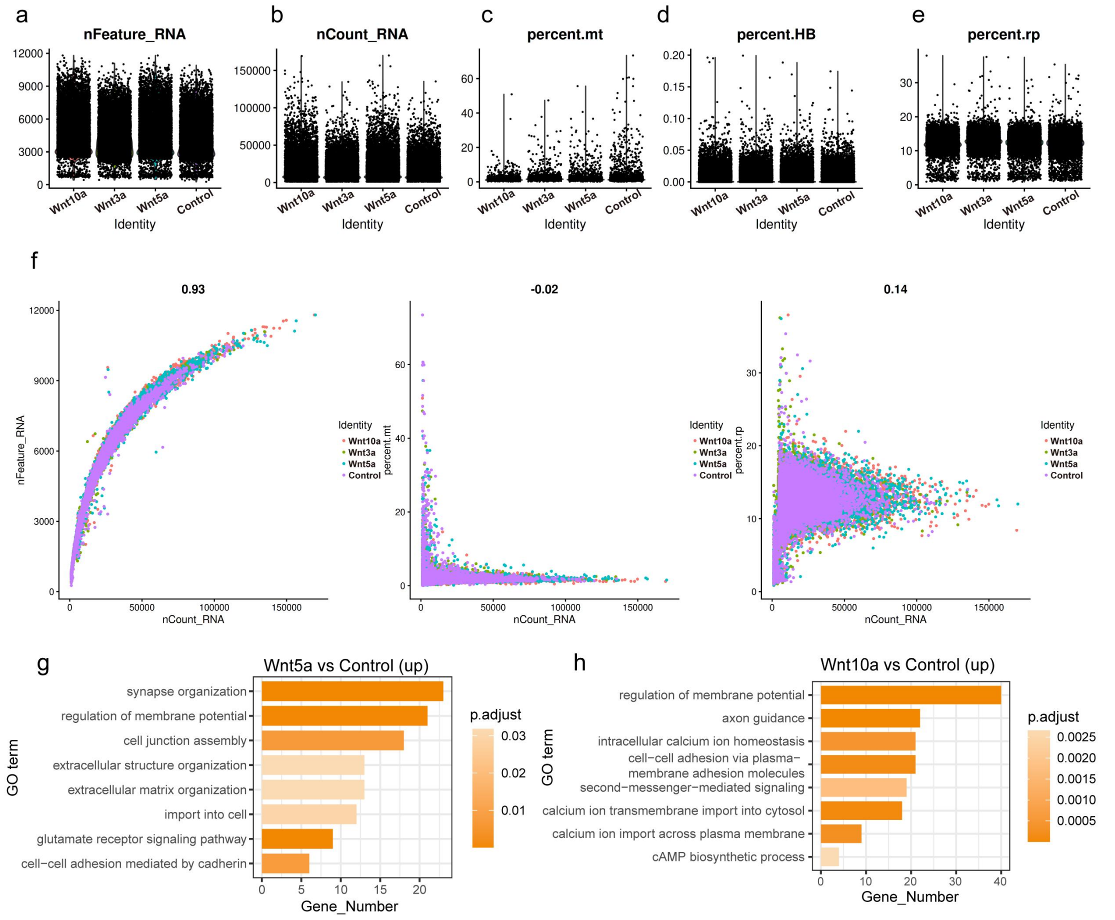

Supplementary figure 1. Single-cell RNA-seq data quality control. (a, b) The distribution plot of gene expression (a) and gene count (b) show that the proportions of potential multiplets and empty cells are relatively low. (c-e) Minimal contamination from mitochondrial genes (c), hemoglobin genes (d), and ribosomal genes (e). (f) Gene expression correlation plot. (g-h) Gene Ontology (GO) enrichment analysis of upregulated DEGs in Wnt5a and Wnt10a-treated groups.

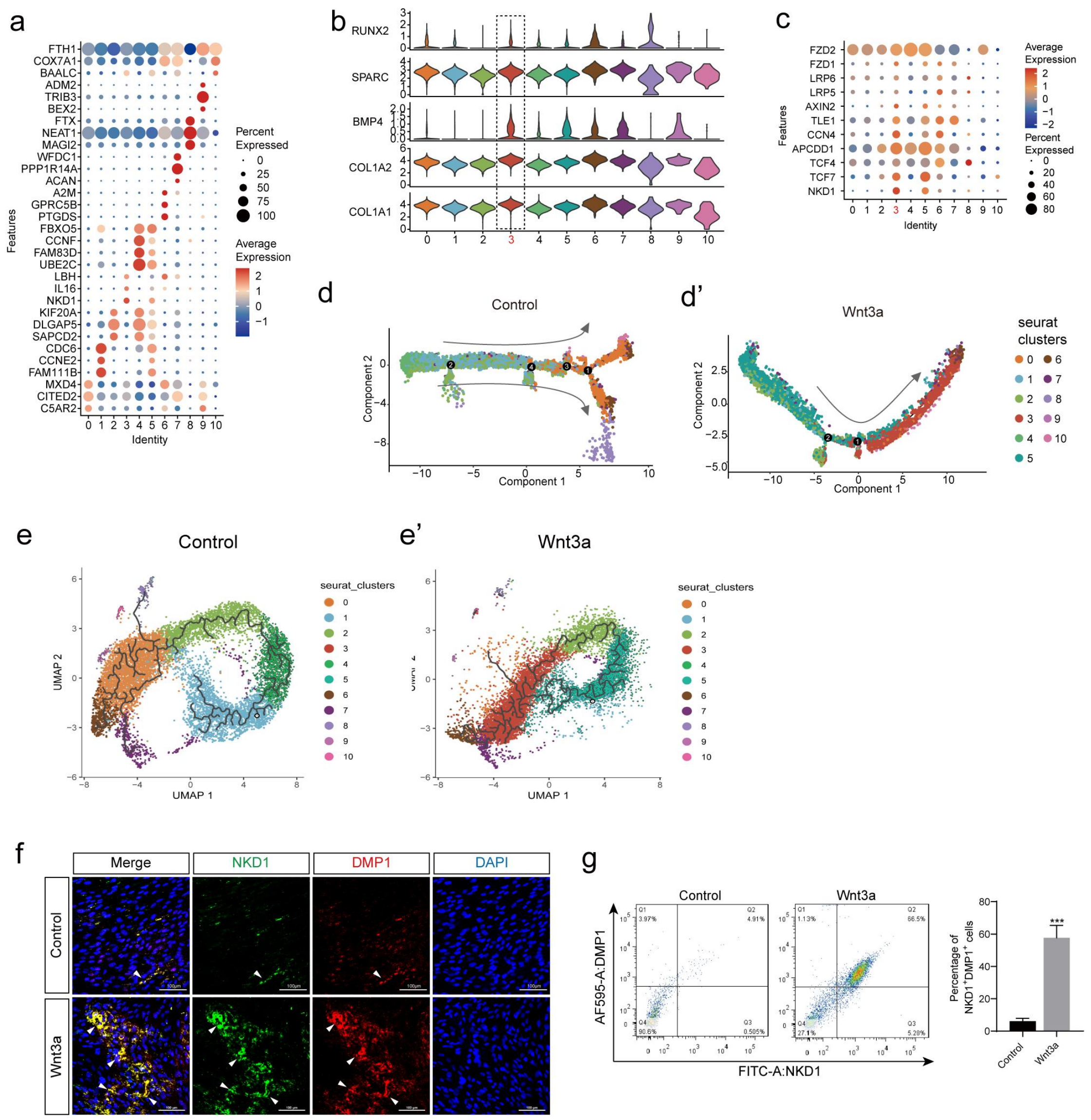

Supplementary figure 2. (a) Dotplot showing marker genes of each cluster. (c) Bar plot showing the proportional distribution of identified clusters. (b) Violin plots showing the expression of several odontogenic differentiation genes. (c) Dotplot showing the expression patterns of canonical Wnt signaling pathway components. (d, d') Monocle2 analysis of Wnt3a-treated and control DPSCs. (e, e') Monocle3 trajectory analysis of Wnt3a-treated and control DPSCs. (f) Immunofluorescence analysis of NKD1 co-localization with DMP1. White arrowheads indicate cells positive for both NKD1 and DMP1. (g) Flow cytometry quantification of $NKD1^{+}DMP1^{+}$cell proportions. (Data are presented as the mean of >3 biological replicates ± SD. ***P < 0.001).

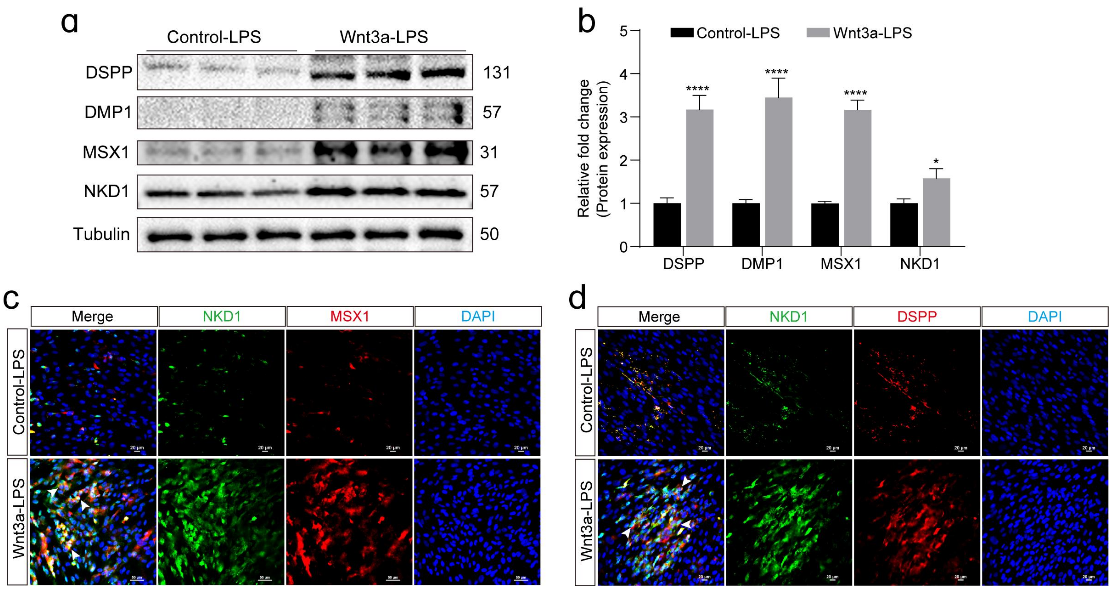

Supplementary figure 3. (a,b) Western blot analysis and quantification showed that Wnt3a induced high expression of NKD1, DSPP, DMP1, and MSX1 under LPS-stimulated inflammatory conditions. (c,d) Under LPS-stimulated inflammatory conditions, Wnt3a induced NKD1⁺ cells with high expression of MSX1 (c) and DSPP (d). (Data are presented as the mean of >3 biological replicates ± SD. *P < 0.05, ****P < 0.0001).

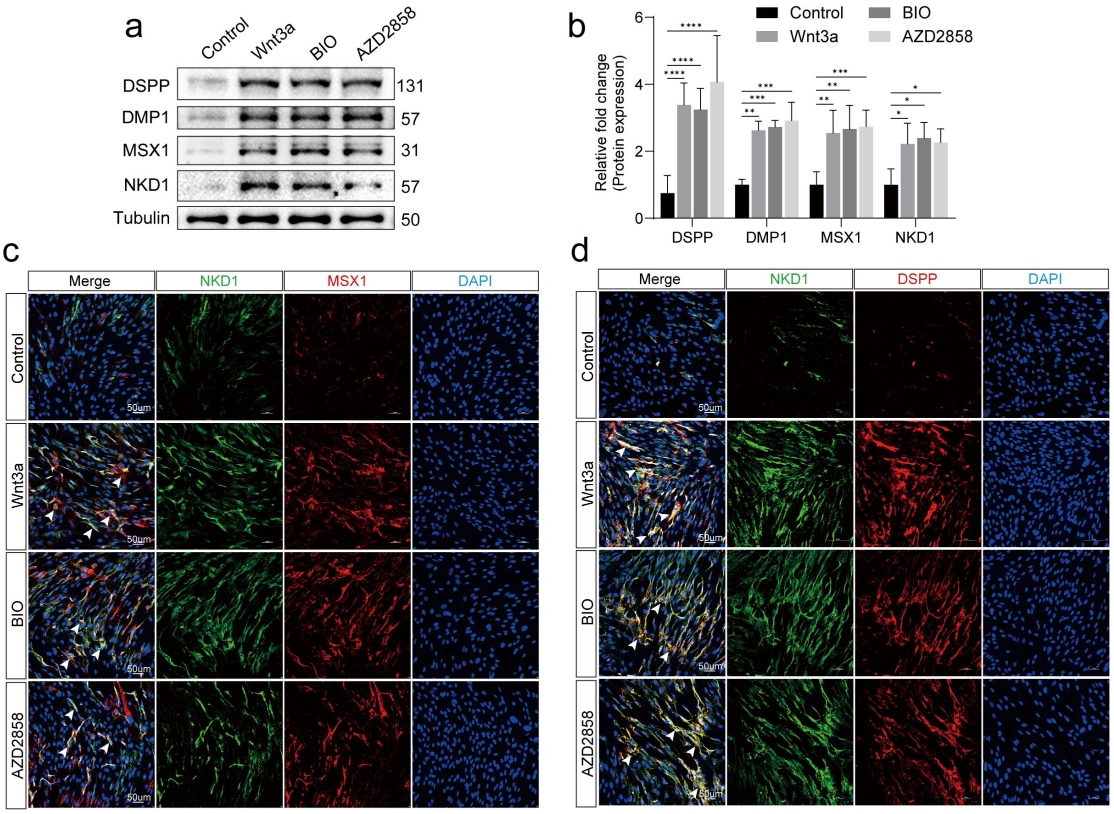

Supplementary figure 4. (a,b) Western blot analysis and quantification demonstrated that Wnt small molecule agonists BIO and AZD2858, similar to Wnt3a, induced high expression of NKD1, DSPP, DMP1, and MSX1. (c,d) Wnt small molecule agonists BIO and AZD2858, similar to Wnt3a, induced NKD1^{+} cells with high expression of MSX1 (c) and DSPP (d). (Data are presented as the mean of >3 biological replicates ± SD. *P < 0.05, **P < 0.01, ***P < 0.001, ****P < 0.0001).

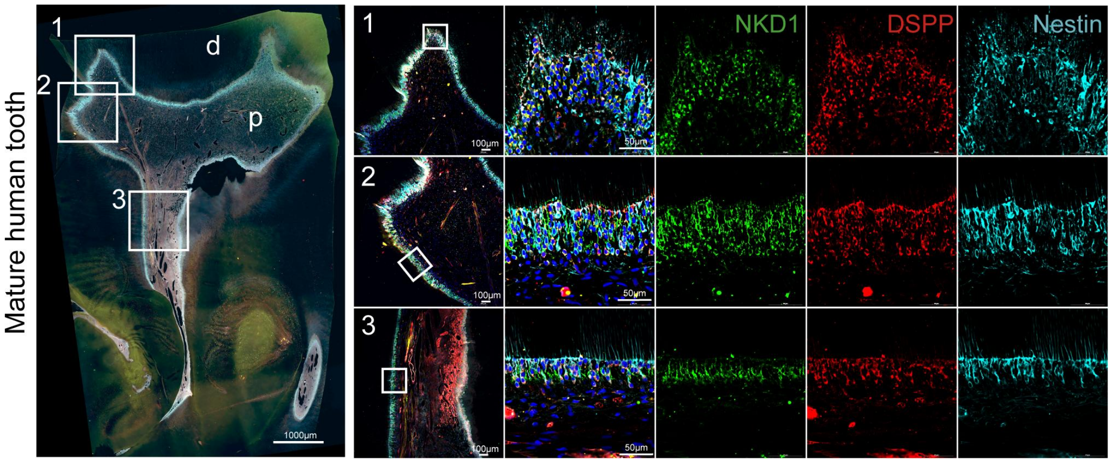

Supplementary figure 5. Immunofluorescence co-localization of NKD1 with DSPP/Nestin in mature human molars. (d, dentin; p, pulp.)

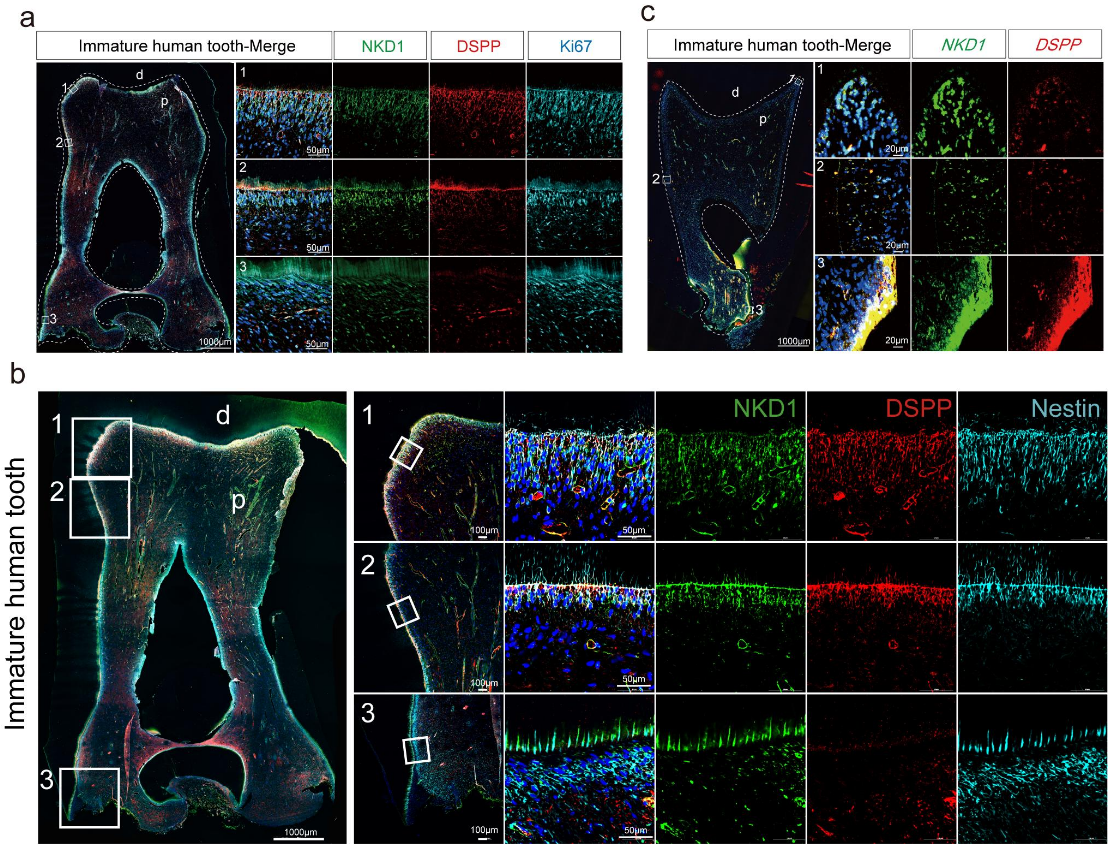

Supplementary figure 6. (a) Immunofluorescence in developing human molars demonstrates NKD1 co-localization with DSPP+Ki67+ odontoblasts in non-apical regions. (b) Immunofluorescence co-localization of NKD1 with DSPP/Nestin in immature human molars. (c) *In situ* hybridization reveals intensified *NKD1/DSPP* co-expression in the apical region of immature molars. (epi, epithelium; mes, mesenchyme; d, dentin; p, pulp.)

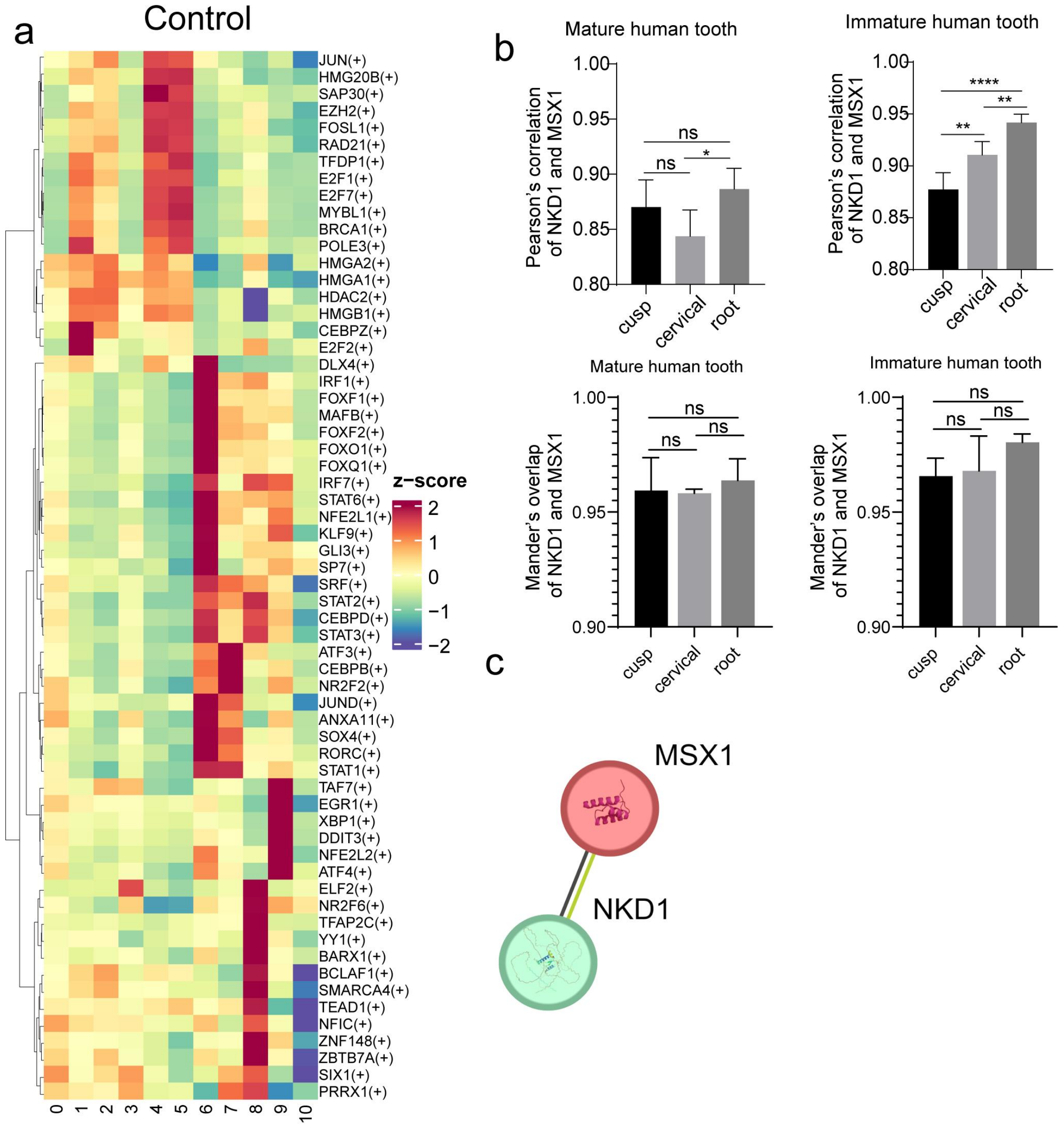

Supplementary figure 7. (a) SCENIC analysis in control samples. (b) Pearson's colocalization coefficients and Mander's overlap coefficients confirm strong NKD1/MSX1 co-localization. (c) STRING analysis predicts functional interaction between NKD1 and MSX1. (ns, no significant difference. Data are presented as the mean of >3 biological replicates ± SD. *P < 0.05, **P < 0.01, ***P < 0.001, ****P <

0.0001).

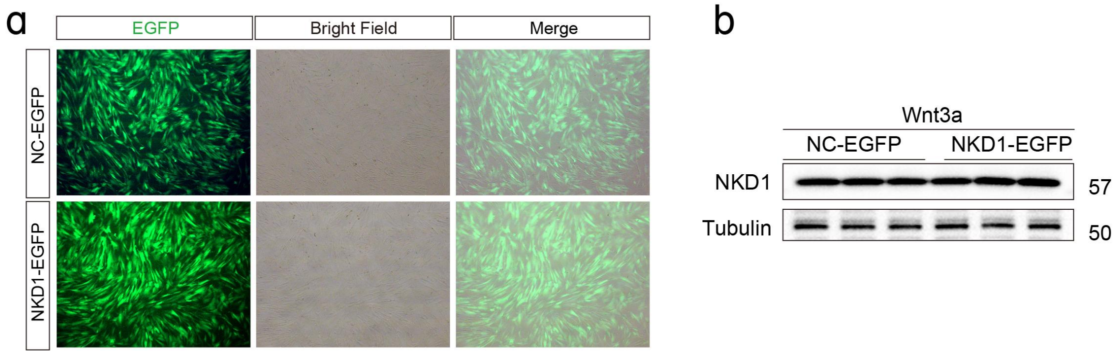

Supplementary figure 8. (a) After NC-EGFP and NKD1-EGFP lentiviral transduction followed by puromycin selection, over 95% of DPSCs were EGFP-positive. (b) NKD1 protein expression levels were equivalent between Wnt3a-treated DPSCs transduced with NC-EGFP and NKD1-EGFP lentivirus.

Table 1: Statistics related to the single-cell raw count matrices.

SampleName	Control	Wnt3a	Wnt5a	Wnt10a
Species	Human	Human	Human	Human
Estimated number of cell	11,668	11,086	9,198	9,408
Mean reads per cell	34,300	31,556	44,067	45,733
Mean UMI count per cell	16,656	17,310	21,081	20,873
Median UMI counts per cell	12,441	13,464	15,467	15,160
Total genes detected	33,215	34,054	34,286	34,460
Mean genes per cell	4,397	4,517	4,945	4,973
Median genes per cell	4,150	4,304	4,725	4,716
Sequencing saturation	44.87%	37.89%	45.62%	48.25%
Fraction Reads in cell	80.87%	83.91%	80.42%	79.80%

Table 2: Information on *In Situ* Hybridization Probes

Serial Number	Name	Sequence (5' to 3')	Modification
1	DSPP	CATGCACCAGGACACCACTTTCTTTGCATCGGAATT	
		ATCCAGGCCAGCATCTTTGGCTCTTCCCAACAGCAT	
		GTGTCTTCTCCTCGGCTACTGCTGTTATTGTCGTGG	

		TTACTGTCACTGCCTTCACTGT	
	Branch Probe H2		
	Signal Probe H2		Cyanine3
2	NKD1-H3	GTGACCTTGCCGTTGTTGTCAAAGTCCTTTACCCGC	
		AGCATCTTGCTGGATGTTTCTCCTCTCGATGTTCTC	
		ATCTACGCGTTGCTGGAGCTCTGAGACCTTGG	
	Branch Probe H3		
	Signal Probe H3		Alexa Fluor 488

Table 3: Primer sequences

Gene	Forward sequence	Reverse sequence
BMP2	TTCGGCCTGAAACAGAGACC	CCTGAGTGCCTGCGATACAG
RHOA	AGCCTGTGGAAAGACATGCTT	TCAAACACTGTGGGCACATAC
PAX9	GGAGGAGTGTTCGTGAACGG	CGGCTGATGTCACACGGTC
SSUH2	AAGGTCCTCCGATGTTTCAGG	ATTTGTGGCATTCCTTGACCA
FAM20C	GGCACAATGCGGAGATTGC	CAGAGCTTCTTGTCCCGTGT
